# Editorial: Imaging in non-small cell lung cancer, volume II

**DOI:** 10.3389/fonc.2024.1412682

**Published:** 2024-04-22

**Authors:** Xin Tang

**Affiliations:** Department of Radiology, Hangzhou Wuyunshan Hospital (Hangzhou Health Promotion Research Institute), Hangzhou, China

**Keywords:** non-small cell lung cancer (NSCLC), radiomics, computed tomography (CT), positron emission tomography (PET/CT), imaging, editorial

Lung cancer is one of the most prevalent malignant tumors with a high incidence and mortality rate. Non-small cell lung cancer (NSCLC) accounts for over 80% of all lung cancers (1). The intricate nature of lung cancer is characterized by its aggressive behavior and complex biological heterogeneity. Imaging plays a crucial role in the diagnosis, differential diagnosis, classification, treatment, and prognosis of lung cancer, especially radiomics, which is visible, reproducible, and quantifies tumor heterogeneity. In this Research Topic “*Imaging in non-small cell lung cancer, volume II*”, we have collected a total of 8 related articles, 4 of which were on PET-related radiomics; 3 of which were on CT-related radiomics; and 1 of which was clinical therapy-related imaging. We are grateful to all the editors, reviewers, and authors who have supported and participated in our work.

Radiomics is a field that extracts a vast array of quantitative features from medical images, transforming static images into dynamic data mines ripe for analysis (2). This burgeoning discipline promises to unveil the hidden patterns within tumors, patterns that the human eye, constrained by its biological limits, may not discern. By coupling radiomics with predictive analytics, researchers and clinicians can now predict disease course, treatment response, and patient prognosis with unprecedented precision. This innovative approach is not just reshaping our understanding of lung cancer but is also redefining the pathways to its diagnosis and treatment (3).

The study by Wang et al. is about testament to this capability, demonstrating how PET/CT radiomics can predict lymphovascular invasion in early-stage NSCLC with remarkable precision. This predictive power opens new avenues for personalized treatment planning, potentially enhancing surgical outcomes and patient survival rates by allowing for more targeted therapeutic interventions based on the specific characteristics of the tumor.

The systematic reviews conducted by Ma et al. and Ge et al. provide compelling evidence of the predictive value of PET/CT radiomics in determining EGFR mutation status in NSCLC patients. This breakthrough underscores radiomics’ potential to guide the utility of targeted therapies, marking a significant advance toward personalized medicine in lung cancer care. Through these detailed insights into tumor biology, radiomics not only improves the precision of treatment but also embodies the future direction of oncology, where therapy is customized to the molecular signature of each patient’s cancer.

Tang et al.‘s review on PET-related imaging radiomics articulates the current and future landscape of this technology in lung cancer. The paper accentuates PET radiomics’ capacity to enhance our diagnostic, prognostic, and predictive tools, despite the existing hurdles such as the need for standardization and broader validation of radiomic features.

The innovative nomogram developed by Xue et al. further exemplifies the strides made in leveraging predictive analytics for lung cancer management. By combining clinical and radiological data, this tool provides a nuanced risk assessment for solitary pulmonary nodules, facilitating early and accurate diagnosis. Such advancements are crucial in the landscape of lung cancer, where early intervention can significantly impact survival rates.

The diagnostic precision that radiomics brings to the table is highlighted by Dong et al.‘s work on differentiating pulmonary MALT lymphoma from pneumonic-type lung adenocarcinoma. By identifying specific CT imaging features, this research offers a pathway to more accurate diagnoses, enabling clinicians to choose the most effective treatment strategies for these distinct conditions.

The application of deep learning and radiomics in predicting the nature of pulmonary ground-glass nodules by Huang et al. shows the cutting-edge intersection of artificial intelligence and medical imaging. This synergy promises to revolutionize lung cancer diagnostics by providing non-invasive, accurate differentiation between benign and malignant nodules, thus potentially sparing patients from unnecessary invasive procedures and accelerating the path to appropriate treatment.

Lastly, equal transformation is the work of Mohammed et al., whose systematic review and meta-analysis shed light on the nuanced impacts of PD-1/PD-L1 inhibitors on clinical imaging and the manifestation of immune-related adverse events (irAEs) in NSCLC patients. This research underscores the critical role of imaging in the era of immunotherapy, not just in tracking tumor response but also in identifying and managing the complex side effects of these powerful treatments. The integration of predictive analytics into this process enhances clinicians’ ability to tailor care to individual patients, optimizing outcomes while minimizing harm.

In conclusion, the contributions within this Research Topic magnify the pivotal role of radiomics and predictive analytics in transforming lung cancer diagnosis and treatment. By mining deep into the wealth of data hidden within medical images, these technologies promise a shift towards more personalized, accurate, and effective cancer care. This promise, however, is not without its challenges. As the studies indicate, the journey towards realizing the full potential of radiomics and predictive analytics is contingent on overcoming hurdles such as the need for standardized methodologies and the broader validation of radiomic features. Despite these challenges, the advancements outlined in this collection of research provide a glimpse into a future where the diagnosis, treatment, and prognosis of lung cancer are significantly improved through the integration of these cutting-edge technologies. The symbiosis between various scientific disciplines and continued innovation is essential, not only for advancing the field, but also for ensuring that the benefits of these technologies can be accessed by all lung cancer patients.

We express our gratitude to all authors and reviewers who contributed to this Research Topic. Their pioneering work not only advances our understanding of lung cancer but also opens new avenues for employing radiomics and predictive analytics in clinical practice. It is our hope that this editorial and the studies it encompasses will inspire further exploration and application of these technologies in oncology, ultimately leading to better patient care and outcomes in lung cancer.

## Author contributions

XT: Conceptualization, Data curation, Writing – original draft, Writing – review & editing.
